# Supplementary material for: Agreement between antenatal gestational age by ultrasound and clinical records at birth: A prospective cohort in the Brazilian Amazon
Source: PLoS One. 2020 Jul 14;15(7):e0236055. doi: 10.1371/journal.pone.0236055 (PMC7360033; doi:10.1371/journal.pone.0236055)
Supplement: S1 File — (PDF) [file pone.0236055.s001.pdf]

Supporting information

Multistage algorithm for assessing the best estimate of gestational age during the antenatal period, considering reliability of last menstrual period and acceptable differences in relation to ultrasound estimates

The present algorithm is based on the following set of variables:

- Gestational age based on last menstrual period (LMP) reported with certainty, in days [GA_LMP];
- Gestational age based on ultrasound examination, in days [GA_US];
- Regularity of menstrual cycles, no or yes [REGULAR_CYCLE, categorized as 0 or 1, respectively];
- Use of hormonal contraceptive methods before conception, no or yes [HORM_CONTRACEP, categorized as 0 or 1, respectively];
- Duration of pregnancy at the ultrasound examination >22 weeks, no or yes [US_22WK, categorized as 0 or 1, respectively];
- Modular difference between GA_US and GA_LMP, in days [DIFF].

Estimates of gestational age based on LMP and ultrasound should refer to a common reference date, covering the same period of time for a given pregnancy. For the ultrasound estimate, a mean value was generated from the measurements of fetal biparietal diameter and femur length, as described in the manuscript. Types of contraceptive methods may include, for instance, condoms, oral contraceptive pills, contraceptive injections, hormonal intrauterine devices and emergency contraceptive pills, in isolate or combined use. Pregnant women who referred no use of contraceptive methods before conception should be combined with those who referred exclusively using non-hormonal methods in the category “no”; pregnant women who referred using any hormonal method before conception, in isolate or combined form, should be categorized as “yes”.

Based on Stata (Stata Corp., College Station, TX, USA) commands, the best estimate of gestational age may be defined step-by-step as follows:

```
generate BEST_GA = GA_US if REGULAR_CYCLE==0
replace  BEST_GA = GA_US if REGULAR_CYCLE==1 & HORM_CONTRACEP==1
replace  BEST_GA = GA_US if REGULAR_CYCLE==1 & HORM_CONTRACEP==0 & US_22WK==0 &
          DIFF>7
replace  BEST_GA = GA_US if REGULAR_CYCLE==1 & HORM_CONTRACEP==0 & US_22WK==1 &
          DIFF>14

replace  BEST_GA == GA_LMP if REGULAR_CYCLE==1 & HORM_CONTRACEP==0 & US_22WK==0 &
          DIFF<=7
replace  BEST_GA == GA_LMP if REGULAR_CYCLE==1 & HORM_CONTRACEP==0 & US_22WK==1 &
          DIFF<=14
```
